# Prognostic value of morphology and hormone receptor status in breast cancer – a population-based study

**DOI:** 10.1038/sj.bjc.6602153

**Published:** 2004-09-07

**Authors:** C Allemani, M Sant, F Berrino, T Aareleid, G Chaplain, J W Coebergh, M Colonna, P Contiero, A Danzon, M Federico, L Gafà, P Grosclaude, G Hédelin, J Macè-Lesech, C M Garcia, E Paci, N Raverdy, B Tretarre, E M I Williams

**Affiliations:** 1 Epidemiology Unit, Istituto Nazionale per lo Studio e la Cura dei Tumori, Via Venezian 1, I-20133 Milano, Italy; 2 Department of Epidemiology and Biostatistics, Institute of Experimental and Clinical Medicine, Hiiu 42, 11619 Tallinn, Estonia; 3 Estonian Cancer Registry, North Estonian Regional Hospital Foundation's Cancer Centre, Hiiu 44, 11619 Tallinn, Estonia; 4 Côte d'Or Breast and Gynaecologic Cancer Registry, Centre Universitaire d'Epidémiologie de population, Faculté de Médicine, 7 blvd Jeanne d'Arc, F-21000 Dijon Cedex, France; 5 Comprehensive Cancer Centre South, Eindhoven Cancer Registry, PO Box 231, NL-5600 AE Eindhoven, The Netherlands; 6 Isère Cancer Registry, 21, chemin des Sources, F-38240 Meylan, France; 7 Lombardy Cancer Registry, Istituto Nazionale per lo Studio e la Cura dei Tumori, Via Venezian 1, I-20133 Milano, Italy; 8 Doubs Cancer Registry, Centre Hospitalo-Universitaire, 2 place Saint-Jacques, F-25030 Besançon Cédex, France; 9 Modena Cancer Registry, Policlinico, Via del Pozzo, 71 I-41100 Modena, Italy; 10 Ragusa Cancer Registry, Via Dante, 109 I-97100 Ragusa, Italy; 11 Tarn Cancer Registry, Recherche en Epidémiologie et Prévention, B.P. 37, F-81001 ALBI Cédex, France; 12 Bas-Rhin Cancer Registry, Laboratoire d'Epidémiologie et de Santé Publique, Faculté de Médecine, 11, rue Humann, F-67085 Strasbourg Cedex, France; 13 Calvados Cancer Registry, Centre François Baclesse, Route de Lion sur Mer, F-14021 Caen Cedex, France; 14 Granada Cancer Registry, Escuela Andaluza de Salud Pública, Campus Universitario de Cartuja, Ap. Correos 2070, E-18080 Granada, Spain; 15 Tuscany Cancer Registry, U.O. di Epidemiologia Clinica e Descrittiva, A.O. Careggi-CSPO, Via di San Salvi, 12 I-50135 Firenze, Italy; 16 Somme Cancer Registry, Association pour la Recherche Epidemiologique en Picardie, C.H.R. Nord, Bât. de Santé Publique, F-80054 Amiens Cedex 1, France; 17 Hérault Cancer Registry, Bât Recherche, Parc Euromédecine, 208, Rue des Apothicaires, F-34298 Montpellier Cedex 5, France; 18 Mersey Cancer Registry, University of Liverpool, 2nd floor, Muspratt Bldg, P.O.Box 147, GB-Liverpool L69 3GB, UK

**Keywords:** breast cancer, population-based studies, relative survival, predictive factors, relative excess risk of death

## Abstract

We analysed the 5-year relative survival among 4473 breast cancer cases diagnosed in 1990–1992 from cancer registries in Estonia, France, Italy, Spain, the Netherlands and the UK. Among eight categories based on ICD-O codes (infiltrating ductal carcinoma, lobular plus mixed carcinoma, comedocarcinoma, ‘special types’, medullary carcinoma, not otherwise specified (NOS) carcinoma, other carcinoma and cancer without microscopic confirmation), the 5-year relative survival ranged from 66% (95% CI 61–71) for NOS carcinoma to 95% (95% CI 90–100) for special types (tubular, apocrine, cribriform, papillary, mucinous and signet ring cell); 27% (95% CI 18–36) for cases without microscopic confirmation. Differences in 5-year relative survival by tumor morphology and hormone receptor status were modelled using a multiple regression approach based on generalised linear models. Morphology and hormone receptor status were confirmed as significant survival predictors in this population-based study, even after adjusting for age and stage at diagnosis.

Tumour morphology and hormone receptor status are established predictors of breast cancer survival (Ruder *et al*, 1989; Northridge *et al*, 1997; Early Breast Cancer Trialists' Collaborative Group, 1998; Li *et al*, 2003). Detailed stage information is seldom available in population-based studies and few have assessed the influence of both these groups of predictors on prognosis using appropriate statistical methods (Northridge *et al*, 1997; Li *et al*, 2003).

The EUROCARE high-resolution studies, which are principally concerned with interpreting regional differences in survival, also provide population-based information on biological prognostic factors, such as tumour morphology and hormone receptor status, as well as information on stage, diagnostic investigations and therapy. Using such data, the present study aimed to examine, at the population level, the influence of morphology and hormone receptor status on breast cancer survival, adjusting by disease stage and age at diagnosis.

## MATERIAL AND METHODS

We analysed data on a representative sample of 4478 breast cancer patients investigated in previous EUROCARE high-resolution studies on breast cancer (Sant, 2001; Sant *et al*, 2003). All had primary invasive breast cancer diagnosed in 1990–1992 in the territories of 17 population-based cancer registries in six countries: Estonia (national registry); France (Bas-Rhin, Calvados, Côte d'Or, Doubs, Hérault, Isère, Somme, and Tarn); Italy (Firenze, Modena, Ragusa and Varese); Spain (Granada); the Netherlands (Eindhoven) and the UK (Mersey and Thames). Details of the study design and sampling have been published (Sant, 2001). At least 5 years of follow-up was available for all women. Each registry used its own follow-up procedures. The UK registries updated the life status of patients included in this study by linking death certificates to the registry records; all other registries actively ascertained the life status of cases from various sources of information (e.g. registry of general practitioners, municipality files).

Detailed information on stage, diagnostic examinations and treatment was collected according to a standardised protocol (Sant, 2001). Most of the information required was obtained from patients' clinical records. However, in some cases these were incomplete, and other records had to be examined (e.g. pathology reports and discharge records).

ICD-O classification (Percy *et al*, 1990) was available for all cases. A total of 4473 cases were included in the morphology analysis after excluding five erroneously classified cases (Table 1

**Table 1:** Total number of breast cancer cases by registry, with data quality indicators

					**Morphology^a^**		**Hormone receptor status^a^**	
**Country**	**Registry**	**No.**	**% Microscopically confirmed**	**% Operated**	**% Infiltrating ductal carcinoma**	**% Carcinoma NOS**	**% Oestrogen available**	**% Progesterone available**
Italy:	Firenze	207	93.7	95.2	49.7	0.5	40.6	34.5
	Modena	302	97.4	95.4	80.2	1.4	70.8	69.1
	Ragusa	217	88.8	87.6	60.0	3.7	65.3	62.1
	Varese	467	96.2	90.8	70.7	1.4	70.5	68.4
								
France:	Bas-Rhin	208	99.5	93.7	82.0	0.0	84.6	84.6
	Calvados	305	98.7	90.5	69.2	6.9	67.7	68.1
	Cote d'Or	251	99.2	93.2	74.4	0.0	89.3	89.3
	Doubs	224	96.4	92.4	73.4	1.9	68.1	67.6
	Herault	202	97.5	89.1	40.6	38.9	68.9	67.2
	Isère	202	98.5	95.5	74.6	4.1	84.5	82.9
	Somme	244	99.2	90.2	36.4	54.5	47.3	46.8
	Tarn	173	97.1	91.9	75.5	3.1	72.3	71.7
								
Spain:	Granada	179	97.2	88.3	77.8	3.2	33.5	33.5
								
UK:	Mersey	219	96.4	87.7	78.1	3.1	0.0	0.0
	Thames	340	94.7	83.2	70.0	10.9	2.1	0.0
								
NL:	Eindhoven	509	98.6	92.7	71.2	1.7	81.4	52.7
	Estonia	224	96.4	74.5	78.4	1.8	28.1	0.0
								
**Total**		**4473**	**96.9**	**90.2**	**68.8**	**7.4**	**59.6**	**53.9**

a Percentages calculated on operated patients.

). Based on the ICD-O code, we grouped the cases into the following eight categories: infiltrating ductal carcinoma, lobular plus mixed carcinoma, comedocarcinoma, the so-called special types (77 tubular, five apocrine, 20 cribriform, 27 papillary, 77 mucinous and five signet ring cell carcinoma) (Tavassoli and Devilee, 2003), medullary carcinoma, not otherwise specified (NOS) carcinoma, other carcinoma (trabecular, acinar, squamous, small cell, pilomatrix, transitional carcinoma, lymphoepithelioma and carcinoid) and cancer without microscopic confirmation.

Oestrogen and progesterone receptor status was classified into five categories: negative, positive, test performed with unknown result, test not performed and unknown whether performed or not. Receptor status, in particular whether positive or negative, was determined directly from the clinical record: absolute values were not required by the study protocol.

Tumours were staged according to TNM rules (3rd edition) (Spiessel *et al*, 1992). Pathological T and N categories were used for women who underwent surgery; clinical T, N and M categories used for women not treated surgically (approximately 10%). We categorised tumour stage as follows: T1N0M0, T2-3N0M0, T1-3N+M0, T4 M0, M1 and stage not specified.

Relative survival (5-year) was calculated by the Hakulinen method (Hakulinen, 1982; Hakulinen and Abeywickrama, 1985) with 95% confidence intervals (CI) calculated from the standard error according to Greenwood's (1926) method. Relative survival is an estimate of the probability of cancer survival after adjusting for competing causes of death determined from general population life tables for women, specific for each country or registry.

Differences in 5-year relative survival by tumour morphology and hormone receptor status were modelled with a recently developed multiple regression approach based on generalised linear models and adopting the Poisson assumption for the observed number of deaths (Dickman *et al*, 2004). The relative excess risks (RERs) derived from these models quantify the extent to which the hazard of death in a given group differs from that in the reference category, after taking into account the background risk of death in the general population of each country or region. The multivariable analyses only included cases from registries with hormone receptor status available for ⩾60% of operated cases (2346 women; 52% of total). Cases from the registries of Firenze (Florence), Somme, Granada, Mersey, Thames, Eindhoven and Estonia were excluded (Table 1).

Age at diagnosis was categorised into <40 years, 40–49, 50–59 (reference category) and 60 years or over. Hormone receptor status was grouped into four categories: both positive (ER+ PGR+), both negative (ER− PGR−), tests performed with one positive result (ER+ or PGR+) and cases with test not performed or result unknown. All calculations were carried out using the Stata statistical package (Stata Corporation, 2001).

## RESULTS

Table 1 shows the total number of cases by registry together with morphology and hormone receptor status and other indicators of data completeness and quality. Overall 97% of cases were microscopically confirmed (range 89–99%) and 90% were treated surgically (range 74–95%). Infiltrating ductal carcinoma and NOS carcinoma accounted for 69 and 7% of the surgically treated cases, respectively, with conspicuous variation across registries (36–82% and 0–54%, respectively). Oestrogen receptor status was available in 60% of operated cases (range 0–89%) and progesterone receptor status was available in 54% of operated cases (range 0–85%).

Table 2

**Table 2:** Number of cases by morphologic group, with 5-year relative survival (all participating registries)

**Morphology**	**No.**	**%**	**5-year relative survival (%)**	**95% CI**
Infiltrating ductal carcinoma	2886	65	81	80–82
Lobular and mixed carcinoma	523	12	86	82–90
Comedocarcinoma	104	2	87	80–94
Special types^a^	211	5	95	90–100
Medullary	62	1	86	76–96
NOS carcinoma	464	10	66	61–71
Other carcinoma^b^	82	2	77	65–89
No microscopic confirmation	141	3	27	18–36
Total	4473	100	79	78–80

a Special types: tubular, apocrine, cribriform, papillary, mucinous, signet ring cell carcinoma.

b Other carcinoma: trabecular, acinar, squamous, small cell, pilomatrix, transitional carcinoma, lymphoepithelioma and carcinoid.

shows the 5-year relative survival by morphology group, for all cases. The largest morphological categories were infiltrating ductal carcinoma (65%), lobular or mixed carcinoma (12%) and NOS carcinoma (10%). The other categories contained many fewer cases. Of all cases, 3% were not confirmed microscopically.

The 5-year relative survival was 81% (95% CI 80–82) for infiltrating ductal carcinoma, 87% for comedocarcinoma (95% CI 80–94), 86% for lobular or mixed carcinoma (95% CI 82–90) and 86% for medullary carcinoma (95% CI 76–96). The special types category had the greatest survival (95%; 95% CI 90–100), NOS carcinoma had the second lowest survival (66%; 95% CI 61–71) and cases without microscopic confirmation the lowest survival (27%; 95% CI 18–36).

Table 3

**Table 3:** Hormone receptor status, with mean age at diagnosis and 5-year relative survival (cases from a subset of 10 registries with information on receptor status available in ⩾60% of operated cases)

	**Oestrogen**					**Progesterone**				
	**No.**	**(%)**	**Mean age**	**5-year relative survival (%)**	**95% CI**	**No.**	**(%)**	**Mean age**	**5-year relative survival (%)**	**95% CI**
Positive	1222	52	61	90	88–92	1091	47	60	90	88–92
Negative	509	22	56	77	73–81	613	26	60	79	75–83
Not available	615	26	62	86	82–90	642	27	62	86	82–90
Total	2346	100	60	86	84–88	2346	100	60	86	84–88

Not available=test not performed or unknown result.

shows hormone receptor status in 2346 cases from a subset of 10 registries with this information available for ⩾60% of operated women, together with mean age at diagnosis and 5-year relative survival. A total of 1222 (52%) of these women had oestrogen-positive cancers, 1091 (47%) had progesterone-positive cancers. More specifically, 980 (42%) had both oestrogen- and progesterone-positive tumours, while 393 (17%) were negative for both oestrogen and progesterone receptors (see Table 4

**Table 4:** Tumour morphology in relation to hormone receptor status for cases with information available on oestrogen (*n*=1731) and progesterone (*n*=1704) from the 10 registries with hormone receptor information available in ⩾60% operated cases

**Morphology**	**Oestrogen positive (%)**	**Progesterone positive (%)**
Infiltrating ductal carcinoma	71	64
Comedocarcinoma	48	50
Lobular and mixed carcinoma	82	70
Special types	69	67
Medullary carcinoma	26	35
NOS carcinoma	64	60
Other carcinoma	66	53

). The mean age of women with oestrogen-positive cancers was slightly greater than for those with oestrogen-negative cancers, but did not differ between those with progesterone-negative and progesterone-positive tumours.

Women with oestrogen receptor-positive disease had better survival (90%; 95% CI 88–92) than those with oestrogen receptor-negative disease (77%; 95% CI 73–81). Similarly, women with progesterone receptor-positive disease had better survival (90%; 95% CI 88–92) than those with progesterone receptor-negative disease (79%; 95% CI 75–83). Women for whom information on receptor status was not available had intermediate 5-year relative survival (86%, 95% CI 82–90). The survival of patients with both receptors positive was 90% (95% CI 87–93) compared to 73% (95% CI 68–78) for those with both receptors negative (data not shown).

Table 4 shows tumour morphology in relation to positive hormone receptor status for the cases with available information on oestrogen (*n*=1731) or progesterone status (*n*=1704) from the 10 registries, with information available in 60% or more of operated cases. The lobular plus mixed carcinoma category had the highest proportions of cases with positive receptor status (82% for oestrogen and 70% for progesterone), while the medullary carcinoma category had the lowest proportions with positive receptors (26% for oestrogen and 35% for progesterone). In the other morphologic groups, the proportions ranged from 48 to 71% for oestrogen and from 50 to 67% for progesterone.

For most morphologic categories, the proportion with positive oestrogen receptors was higher than the proportion with positive progesterone receptors, exceptions being comedocarcinoma and medullary carcinoma.

Table 5

**Table 5:** Relative excess risks (RERs) of death by morphology, age, disease stage and hormone receptor status (cases from the 10 registries with hormone receptor information available in ⩾60% operated cases)

		**No of cases**	**RER**	**95% CI**
Morphology	Infiltrating ductal carcinoma	1659	1	
	Lobular and mixed carcinoma	297	0.58	0.37–0.91
	Comedocarcinoma	66	0.34	0.12–0.98
	Special types	111	0.35	0.12–0.99
	Medullary carcinoma	34	1.19	0.49–2.85
	NOS carcinoma	130	1.08	0.68–1.71
	Other carcinoma	49	1.38	0.72–2.63
				
Age	23–39	144	0.96	0.60–1.53
	40–49	426	0.71	0.49–1.02
	50–59	486	1	
	60–99	1290	0.86	0.64–1.14
				
Stage	T1N0M0	819	1	
	T2-3N0M0	443	3.82	1.94–7.51
	T1-3N+M0	789	9.26	5.05–16.97
	T4 M0	115	17.78	9.07–34.83
	M1	72	56.39	29.42–108.09
	Stage not specified	108	6.04	2.42–15.11
				
Hormone receptors	ER− PGR−a	393	1	
	ER+PGR+^b^	980	0.32	0.24–0.43
	Either ER+ or PGR+^c^	353	0.41	0.28–0.62
	Other^d^	620	0.49	0.35–0.67

a ER− PGR−=both negative results.

b ER+ PGR+=both positive results.

c Either ER+ or PGR+=tests performed with one positive result; in most cases, it is not known whether the other test was performed.

d Other=tests not performed or unknown result.

shows the results of the multivariable regression analysis to determine the RERs of death for morphologic group, age, stage at diagnosis and hormone receptor status, in each case taking into account the influence of the other variables.

Comedocarcinoma, (0.34, 95% CI 0.12–0.98), special types (0.35, 95% CI 0.12–0.99) and lobular and mixed carcinoma (0.58, 95% CI 0.37–0.91) had lower RERs of death than infiltrating ductal carcinoma (reference category). For the other carcinomas category, the RER (1.38, 95% CI 0.72–2.63) was about 40% higher and for the medullary carcinoma category the RER (1.19, 95% CI 0.49–2.85) was about 20% higher than reference; the RER for NOS carcinoma was 1.08 (95% CI 0.68–1.71). With respect to age, the 40–49 year age group had the lowest RER of death (0.71, 95% CI 0.49–1.02) compared to the 50–59 year reference category. With respect to tumour stage, RERs increased with advancing stage and in all cases were significantly higher than reference (T1N0M0). Unspecified stage cancers had an intermediate RER.

Women positive for both hormone receptors (ER+ PGR+) had a significantly lower RER of death (0.32, 95% CI 0.24–0.43) than those negative for both receptors (ER− PGR−, reference). A positive result for one receptor (ER+ or PGR+) was also significantly protective compared to reference (0.41; 0.28–0.62). Women with receptors not determined or with unknown result had an intermediate RER (0.49, 95% CI 0.35–0.67). In a separate analysis of the only ER+ and only PGR+ groups, RERs were 0.39 (95% CI 0.24–0.62) and 0.51 (95% CI 0.27–0.97), respectively, compared to the ER- PGR- reference (data not shown).

We also tested the two-way interactions: morphology and stage, morphology and age, morphology and registry, hormone receptor status and registry. None of these interactions was statistically significant.

## DISCUSSION

As in previous population-based and clinical studies (Ruder *et al*, 1989; Northridge *et al*, 1997; Early Breast Cancer Trialists' Collaborative Group, 1998; Li *et al*, 2003), tumour morphology and hormone receptor status are important predictors of breast cancer survival. The 5-year survival varied from 66% for NOS carcinoma to 95% for special type tumours, while patients with oestrogen- and progesterone-positive tumours had better survival (90%) than those negative for both receptors (73%). Morphology and receptor status remained significant survival predictors after adjusting for age and stage at diagnosis.

A strength of our study is that it is population-based (covering all the cases in registry populations) and is therefore not susceptible to the selection bias of clinical series. Detailed stage at diagnosis not usually available in population-based studies and age were taken account of in analysing the prognostic effects of morphology and receptor status.

Previous studies on the present data set analysed the effect of tumour stage on survival (Sant *et al*, 2003, 2004). We used this detailed stage information – available in 92% of cases – to adjust for stage in the multivariable analysis of prognostic effects of morphology and receptor status. Although stage depends on the number of axillary nodes examined, this number was similar for each morphological group; thus, inclusion of number of nodes examined in the model had little effect on the results (data not shown).

Another strength of the study is that the effects of competing causes of death were controlled for by estimating the *relative* survival. Population-based studies on the SEER database, which analysed the relative risk of breast cancer death according to tumour morphology, did not estimate survival in this way. One of these (Li *et al*, 2003) did not control for competing causes of death; the other (Northridge *et al*, 1997) used *cause-specific* survival to control for competing causes of death. It is also important to note that only summary information on stage was available in these studies (categories: localised disease, regional metastasis, distant metastasis and unstaged) and the models did not adjust for hormone receptor status.

Information on tumour morphology was satisfactorily complete in the present study (97% of diagnoses confirmed microscopically). The morphology categories special type and comedocarcinoma were associated with lower RERs of death than infiltracting ductal carcinoma (Table 5). The special type category includes, tubular, mucinous and papillary histotypes, which were reported to have good prognoses in a previous population-based study (Li *et al*, 2003), and in other studies (Cooper *et al*, 1978; Fisher *et al*, 1980; Wargotz and Silverberg, 1988; Ellis *et al*, 1992; Silverstein *et al*, 1994; Pedersen *et al*, 1995; Reinfuss *et al*, 1995; Toikkanen *et al*, 1997; Diab *et al*, 1999). Comedocarcinoma also had a relatively good prognosis in a previous study (Li *et al*, 2003). As expected, the categories other and NOS carcinoma had the lowest survival in our study.

Adjustment for age and stage had only limited effects on RERs for each morphologic category; in particular, rank did not change. However, comedocarcinoma, other and NOS carcinoma were generally diagnosed at a more advanced stage than other morphologic categories, and their RERs decreased from 0.55 to 0.34, from 1.57 to 1.38 and from 1.16 to 1.08, respectively, after these adjustments (data not shown). The overall proportion of NOS carcinomas was 7%, compared to 5% in the US SEER populations (Northridge *et al*, 1997).

A possible weakness of our study is that the morphologic diagnoses may not be entirely comparable between registries, as is suggested by the fact that, although all registries coded tumour morphology using ICD-O, the proportion of NOS carcinomas varied considerably between registries (Table 1), indicating that the quality of morphology data also varied. Thus, in Firenze, the proportions of NOS and infiltrating ductal carcinoma were strikingly low because pathologists systematically search for lobular or other components, so that a proportion of these cases are classified as mixed (lobular plus ductal) or special type carcinoma. Only one registry with a high proportion of NOS carcinomas (Herault, 39%) was included in the multivariable analysis, while the average proportion of NOS carcinomas in the other nine registries was around 2%. To identify misclassification in morphology coding, we looked for an interaction between morphology and registry, but found none. In addition, sensitivity analyses excluding Herault and Somme did not produce substantial changes in morphology RERs. We grouped tubular, apocrine, cribriform, papillary and mucinous carcinomas into the special type category because of their small numbers and because all had similarly favourable survival. Larger studies may be able to assign meaningful individual RERs to these rarer histotypes.

We are aware of no other studies that considered the prognostic influence of receptor status on the prognostic effect of morphology. Receptor status is not usually available to cancer registries, and for various reasons registries may not be able to obtain this information systematically. The percentage of our cases with known receptor status was lower than reported elsewhere (Early Breast Cancer Trialists' Collaborative Group, 1998; Chu *et al*, 2001; Chu and Anderson, 2002; Colditz *et al*, 2004). To enhance its reliability, our analysis was confined to registries with receptor status information available in at least 60% of cases. In these registries, the proportions of cases positive for oestrogen and progesterone receptors were similar to those of these other studies.

It is relevant that we do not know the criteria for deciding whether a tumour was receptor positive or negative, or to what extent these were uniform across our registry areas, a problem that has been addressed elsewhere (Chapman *et al*, 1993, 1996). We decided to collect the results as positive or negative. However, this may have reduced accuracy, as we did not have the absolute values against which to check the positive/negative result. To mitigate this problem, we erected four particular categories: ER+ plus PGR+; ER− plus PGR−; either ER+ or PGR+ (with the other result very often not known); and ‘other’ (cases with status unavailable or unreliable). The first two categories are likely to contain the least errors. As expected, ER+ plus PGR+ cases had better prognoses than ER− plus PGR− cases; either ER+ or PGR+ cases also had good prognoses, but less so than cases with both receptors present. The RER of death for the ‘other’ category was intermediate, suggesting that it contained considerable numbers of women whose tumours were receptor positive. Like those for morphology, RERs for receptor status did not change substantially when adjusted by stage and morphology (not shown).

We did not include tamoxifen treatment in the multivariable analysis, as information was incomplete, being often prescribed during follow-up, and not entered on the primary treatment record. However, we did analyse tamoxifen in all ER+ women, since for most of these hormone treatment information was available. Tamoxifen was found to be associated with a 10% reduction in the RER of death compared with ER+ women not prescribed the drug (results not presented) – a figure consistent with reductions reported elsewhere (Early Breast Cancer Trialists' Collaborative Group, 1998). We note that women in the 40–49 age class (predominantly premenopausal) had the lowest RER of death, consistent with previous findings (Langlands and Kerr, 1979; Adami *et al*, 1986; Sant *et al*, 2003).

Our analysis has shown that EUROCARE high-resolution studies are able to provide information on biological tumour characteristics at the level of the population, in addition to their principal aim of explaining regional and temporal survival differences. It is important to continue efforts to improve the completeness and quality of the data collected by population-based cancer registries.
